# Biomechanical study of proximal adjacent segment degeneration after posterior lumbar interbody fusion and fixation: a finite element analysis

**DOI:** 10.1186/s13018-019-1150-9

**Published:** 2019-05-15

**Authors:** Shuai Jiang, Weishi Li

**Affiliations:** Department of Orthopaedics, Peking University Third Hospital, No. 49 North Huayuan Road, Haidian District, Beijing, 100191 China

**Keywords:** Adjacent segment degeneration; Finite element analysis, Range of motion, Intervertebral disc pressure

## Abstract

**Purpose:**

To investigate the biomechanical changes in the proximal adjacent segment with different grades of degeneration after posterior lumbar interbody fusion (PLIF).

**Methods:**

We created three finite element models of the L3–5 with different grades of degeneration (healthy, mild, and moderate) at the L3–4 that were developed by changing the disc height and material properties of the nucleus pulposus. The L4–5 were operated by interbody fusion cages and pedicle screws. All models were loaded with a compressive pre-load of 400 N and a bending moment of 10 N a in three planes to recreate flexion, extension, lateral bending, and axial rotation. The range of motion (ROM), nucleus pressure, and annulus fibrosus pressure of the L3–4 were evaluated.

**Results:**

The ROM, nucleus pressure, and annulus fibrosus pressure increased at the L3–4 after PLIF. As the degeneration progressed in the L3–4, the ROM of the L3–4 decreased while the nucleus pressure and annulus fibrosus pressure increased.

**Conclusions:**

Adjacent segment degeneration (ASD) may be related to the ROM and intradiscal pressure after PLIF. However, as the degeneration of the proximal adjacent segment increases, the ROM in the proximal adjacent segment gradually decreases, but the pressure on the nucleus pulposus and annulus fibrosus gradually increases. Degeneration of the proximal adjacent segment before operation is a risk factor for the ASD after PLIF.

## Background

The number and the rate of spinal fusion surgeries have been increasing annually [1, 2]. One study reported that the annual number of lumbar spinal fusion performed in the USA has rapidly increased by 2.7 times during the past decade [3]. Recently, adjacent segment degeneration (ASD) has become a major concern after fusion surgery. ASD refers to degenerative changes in the unfused segment adjacent to the fusion segments after lumbar fusion, which may lead to the recurrence of lower back pain and radiculopathy. This affects the long-term efficacy of lumbar fusion surgery and even leads to reoperation in some patients. Due to the different diagnostic criteria and follow-up times, the incidence of ASD reported in different studies varies widely, ranging from 5 to 77% [4]. After the lumbar fusion surgery, up to 20% of patients may experience the recurrence of symptoms due to ASD and even require reoperation [5]. Moreover, the success rate of reoperation is much lower than that of the initial surgery [6].

Posterior lumbar interbody fusion and fixation is widely used in lumbar degenerative diseases. Compared with non-fixation fusion surgery, pedicle screws provide stronger fixation and higher fusion rates. Lumbar pedicle fixation and interbody fusion alter the biomechanics of the entire lumbar spine and may accelerate the degeneration of adjacent segments. The relationship between ASD and lumbar fusion surgery has been discussed in several reports [7, 8]; however, there is no definitive knowledge about the biomechanics or risk factors.

Although the exact mechanism is not yet clear, changes in biomechanics after lumbar fusion fixation play an important role in the development of ASD. The area of adjacent segment biomechanical forces and motion has been studied since the 1980s [9, 10]. In 1984, Lee and Langrana [9] found, through in vitro mechanical experiments, that the activity of the adjacent segment and the intervertebral disc pressure were significantly increased after lumbar spinal fusion fixation. Chen et al. [11] discovered that the intervertebral disc pressure (IDP) in adjacent segments after lumbar fusion fixation was increased and that the pressure in the proximal segment was increased more significantly than that in the distal segment. The degeneration of the intervertebral disc itself also has a large impact on the lumbar biomechanics. Kettler et al. [12] found that the early degeneration of the intervertebral disc in vitro resulted in a decrease in the ROM of lumbar spine flexion, extension, and lateral bending. Rohlmann et al. [13] reported that degenerated intervertebral discs increase the maximum von Mises stress of the annulus fibrosus matrix. Axelsson et al. [14] found increased mobility occurring in adjacent segments after L4–5 fusion surgery in 1/3 of the patients. In an in vivo model, Hayes et al. [15] found increased translational motion in adjacent segments when L3–4 was fused, and this motion correlated with low back pain.

Although lumbar fusion fixation and disc degeneration have a significant impact on lumbar biomechanics, there are few reports on how the biomechanical properties influence the degeneration of the adjacent segment after fusion fixation. Biomechanical changes in adjacent discs with different degrees of degeneration after fusion fixation are of particular interest. This study established a lumbar fusion fixation model with three different degrees of proximal ASD. Our aim was to investigate the changes in intervertebral motion and IDP with the progression of proximal degeneration of the lumbar spine after fusion surgery.

## Methods

### FE modeling

**Fig. 1:**
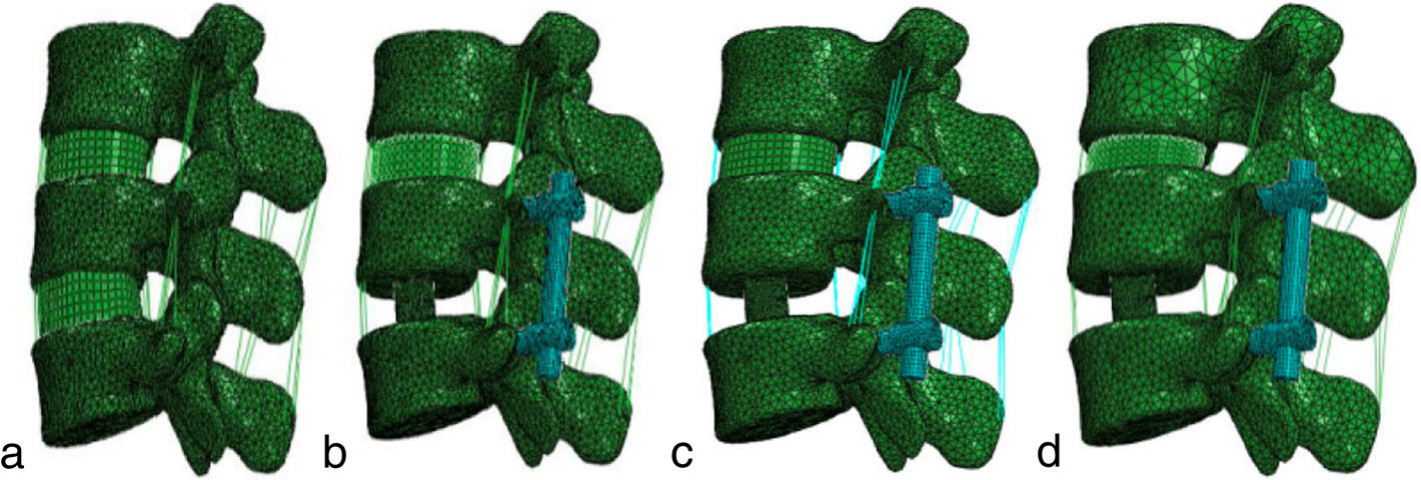
Four finite element models of the L3-L5 lumbar spine (**a**) Healthy disc with no fusion (**b**) Healthy disc with L4-L5 posterior lumbar interbody fusion (**c**) Mild degenerated disc and L4-L5 posterior lumbar interbody fusion (**d**) Moderate degenerated disc and L4-L5 posterior lumbar interbody fusion

A healthy male volunteer was recruited. He had no previous spinal disease or lower back pain. A healthy L3–5 lumbar nonlinear finite element model was established using scanned computed tomography images of the lumbar region. The vertebral body consisted of cancellous bone, cortical bone, endplates, and posterior bone, and the thickness of the cortical bone and endplate was 1 mm. Intervertebral discs were consisted of a nucleus pulposus, an annulus fibrosus matrix, and annulus fibrosus fibers. The fluid traits of the annulus fibrosus matrix and nucleus pulposus were modeled using Mooney-Rivlin hyperelasticity [16]. The contact between the facet joints of the vertebral body was similar to that of facial contact without friction. The anterior longitudinal ligament, posterior longitudinal ligament, ligamentum flavum, interspinous ligament, supraspinous ligament, intertransverse ligament, and annulus fibers were all simulated with uncompressed truss elements [16, 17]. Three models of L3–4 intervertebral discs were established with different degrees of degeneration (normal, mild degeneration, and severe degeneration). The L3–4 intervertebral disc degeneration models were established by changing the height of the intervertebral disc, where the mildly degenerated and severely degenerated disc heights were reduced by 16.5% and 33%, respectively [7]. Nuclear parameters associated with mild and severe degeneration were obtained from the work of Guo et al. [16]. The L4–5 posterior interbody fusion model was simulated using pedicle screws, interbody fusion cages, and grafts placed inside the cage (Fig. 1). The material properties used in the models were obtained from the literature [16–18] (Table 1). The mesh refinement was locally controlled at the highly stress-concentrated sites and the articulating surfaces. Using aspect ratio and the Jacobian check, the quality of all elements was monitored to avoid sharp discontinuities and unrealistically high-stress concentrations. Mesh refinement was performed for modeling accuracy until excellent monotonic convergence behavior with less than 3% difference in the total strain energy was achieved. Healthy disc with no fusion lumbar model has a total of 137,700 elements. Healthy disc with the fusion model has a total of 359,016 elements. Mild degenerated disc and fusion model have a total of 353,471 elements. Moderate degenerated disc fusion model has a total of 368,912 elements.

**Table 1:** Material properties used in the finite element models

Materials	Young’s modulus (MPa)	Poisson’s ratio	Cross-sectional area (mm^2^)	Element type
Vertebra				
Cortical bone	120,000	0.3	–	S3
Cancellous bone	100	0.2	–	C3D4
Posterior bone	3500	0.25	–	C3D4
Endplate	23.8	0.4	–	S3
Intervertebral disc				
Annulus ground substance (Mooney-Rivlin law)	*C*_10_ = 0.18 *C*_01_ = 0.045	–	–	C3D8
Nucleus pulpous (Mooney-Rivlin law)	*C*_10_ = 0.12 *C*_01_ = 0.03 (healthy) *C*_10_ = 0.14 *C*_01_ = 0.035 (mild) *C*_10_ = 0.17 *C*_01_ = 0.041 (moderate)	–	–	C3D8
Annulus fiber layers	360–550	0.3	0.7	T3D2
Ligaments				
Anterior longitudinal	7.8 (< 12.0%), 20 (> 12.0%)	0.3	63.7	T3D2
Posterior longitudinal	10.0 (< 11.0%), 20 (> 11.0%)	0.3	20	T3D2
Ligamentum flavum	15.0 (< 6.2%), 19.5 (> 6.2%)	0.3	40	T3D2
Supraspinous	8.0 (< 20.0%), 15 (> 20.0%)	0.3	30	T3D2
Interspinous	10.0 (< 14.0%), 11.6 (> 14.0%)	0.3	40	T3D2
Intertransverse	10.0 (< 18.0%), 58.7 (> 18.0%)	0.3	1.8	T3D2
Capsular	7.5 (< 25.0%), 32.9 (> 25.0%)	0.3	30	T3D2

*S3* 3-node triangular element, *C3D4* 4-node tetrahedral element, *C3D8* 8-node hexahedral element, *T3D2* 2-node truss element

### Boundary and loading conditions

We limited the movement of all models in all directions of the lower endplate of the L5 vertebral bodies. We applied 400 N of vertical downward pressure to the upper endplate of the L3 vertebral bodies to simulate the body’s own weight [17]. On the L3 upper endplates, bending moments of 10 N O were applied to simulate the movement of the human body in six directions [17] (flexion-extension, lateral bending, and torsion).

### Model validation

We measured the range of motion (ROM) of the L3–4 and L4–5 vertebrae under a 400 N load and 10 N l bending moment using the L3–5 model. The model was verified by comparison with previous experimental data obtained from corpses.

### Measurement data

The ROM, nucleus pressure, and annulus pressure of L3–4 were measured. All finite element analyses were performed using the ABAQUS software, version 6.13 (Dassault Systtult Simulia Corp. Providence, Rhode Island, USA).

## Results

### Validation of the FE model

The healthy L3–5 lumbar finite element model was compared with the previous cadaveric experimental data. Compared with the previous cadaveric experimental data, the L3–4 and L4–5 ROMs derived from the lumbar finite element model were similar to those in a previous cadaver experiment (Table 2), thereby validating the model.

**Table 2:** Ranges of motion predicted by the intact FE model compared with reported ROMs from in vitro study

L3–4	Flexion	Extension	Lateral bending	Torsion (Left)
Yamamoto	7.5 ± 0.8	3.7 ± 0.3	5.7 ± 0.3 (Left) 5.8 ± 0.5 (Right)	2.7 ± 0.4 (Left) 2.5 ± 0.4 (Right)
Present study	8.11	3.97	5.61	2.30
L4–5	Flexion	Extension	Lateral bending	Torsion
Yamamoto	8.9 ± 0.7	5.8 ± 0.4	5.5 ± 0.5 (Left) 5.9 ± 0.5 (Right)	1.7 ± 0.3 (Left) 2.7 ± 0.5 (Right)
Present study	8.26	6.02	5.14	2.69

The lateral bending and torsion values in the present study are expressed as averages

### L3–4 ROM associated with different grades of degeneration

**Fig. 2:**
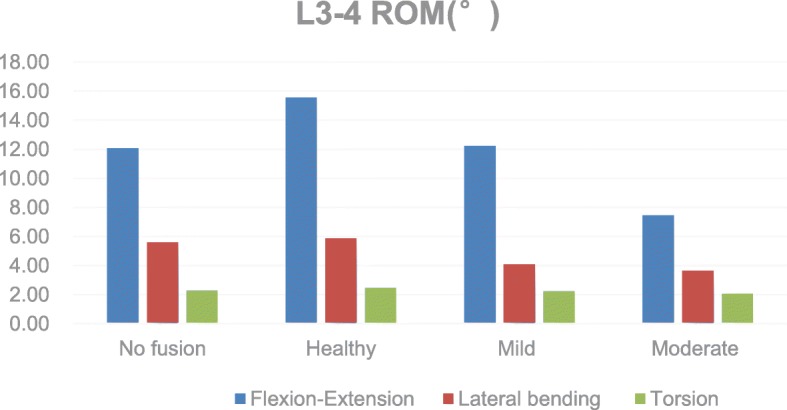
Comparison of the ROM for the L3-L4

We found that after L4–5 PLIF, the L3–4 ROM increased by 34.2%, 4.6%, and 7.8% in flexion-extension, lateral bending, and rotation, respectively. However, in the degeneration models, the L3–4 ROM was significantly reduced compared with that in the model without degeneration. In mild degeneration,the ROM decreased by 24.5%, 30.5%, and 9.7%. In severe degeneration, the ROM decreased by 54.0%, 38.0%, and 16.9% (Fig. 2).

### L3–4 nucleus pulposus and annulus fibrous pressures associated with different grades of degeneration

**Fig. 3:**
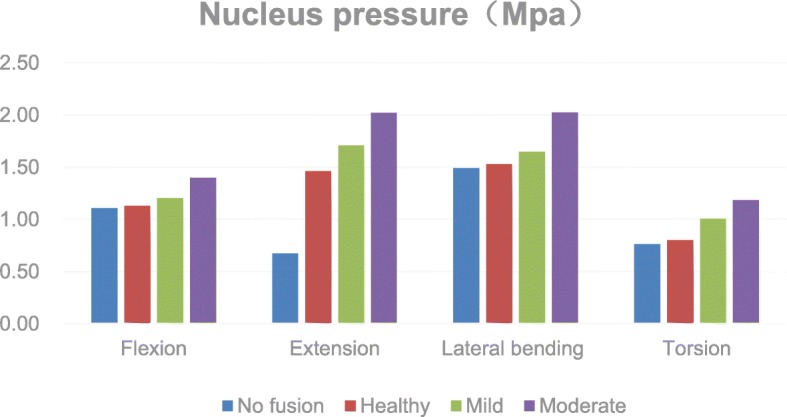
Comparison of the pressure for the L3-L4 nucleus

The pressure on the L3–4 nucleus pulposus after PLIF was increased by 2.2%, 32.7%, 2.7%, and 5.1% in flexion, extension, lateral bending, and rotation, respectively. The pressure on the nucleus pulposus increased further with the degeneration of adjacent segments. In mild degeneration, the nucleus pulposus pressures increased by 6.4%, 16.5%, 7.6%, and 25.4%. In severe degeneration, the nucleus pulposus pressure increased by 23.5%, 38.1%, 32.3%, and 47.8% (Fig. 3).

**Fig. 4:**
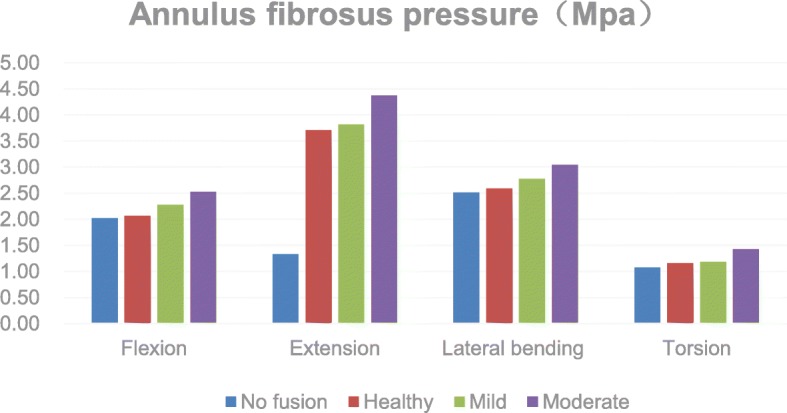
Comparison of the pressure for the L3-L4 annulus fibrosus

The pressures increased on the L3–4 annulus fibrous after PLIF were increased by 1.9%, 176.0%, 2.8%, and 8.0% in flexion, extension, lateral bending, and rotation, respectively. The pressures on the annulus fibrosus increased further with the degeneration of adjacent segments. In mild degeneration, the annulus fibrosus pressure increased by 10.3%, 2.8%, 7.4%, and 1.9%. In severe degeneration, the annulus fibrosus pressure increased by 22.5%, 17.8%, 17.6%, and 22.6% (Fig. 4).

## Discussion

Spine surgeons increasingly choose lumbar posterior fusion fixation to treat lumbar degenerative diseases that are ineffectively cured by conservative treatment. However, the higher incidence of ASD after fusion has limited the long-term outcome after surgery. Although the definitive mechanism of ASD is not fully clarified, previous biomechanical studies indicated that the increases in the ROM and the IDP of adjacent segments are the most likely causes [19]. The biomechanical properties of the discs are also altered according to the degree of disc degeneration, which mainly affects the intervertebral motion and pressure on the intervertebral disc. Nevertheless, the ROM and IDP of adjacent segments with different degrees of degeneration after fusion fixation are rarely reported.

Weinhoffer et al. [20] found that in flexion at the same angle in vitro, the pressure in the proximal segment in the fusion group was higher than that in the unfused group. Cunningham et al. [21] showed in vitro that the pressure in adjacent segments increased by 45% at 12.5segment in the fust al. [22], through finite element analysis, demonstrated that after L3–4 fusion fixation, the IDP in the proximal L2–3 segment was significantly increased in all directions of motion. Figures 3 and 4 of this paper show that in flexion and extension, lateral bending, and rotation, the pressures on the annulus fibrosus and the nucleus pulposus are increased with the most significant increase in posterior extension. This result is consistent with those of previous reports. We found that the increase in the pressure on the annulus fibrosus and nucleus pulposus was most pronounced during extension, at 176.0% and 32.7%, respectively. As the pressure on the adjacent segment of the intervertebral disc increases, over time, this will inevitably lead to the accelerated degeneration of the intervertebral disc. This suggests that the possibility of excessive extension after fusion fixation may be more closely related to the progression of ASD.

Sim et al. [23] performed an L4–5 fixation fusion on 14 fresh cadavers and simulated a biomechanical analysis of flexion, extension, rotation, and lateral bending. The results showed that the ROM of adjacent segments increased after posterior lumbar interbody fusion or transforaminal lumbar interbody fusion. In in vitro experiments in dogs, Ha et al. [24] found that the ROM of the adjacent segments after fusion fixation was increased by 62%, 85%, 30%, and 26% in the flexion, extension, left bending, and right bending, respectively. Through finite element analysis, Park et al. [25] found that the ROM of the proximal adjacent segment increased by 11.1–33.8% in the flexion-extension direction after single segment fusion fixation. The greater the segment fixation, the higher the increase in the activity of adjacent segments. Figure 2 shows that the ROM of adjacent segments after fusion fixation was increased in all directions; in particular, there was a 34.2% increase in the flexion-extension direction. The increase in the ROM of the adjacent segment may be due to an increase in IDP. As the pressure increases, the intervertebral disc undergoes increased deformation, resulting in increased intervertebral motion. The activity of flexion and extension may be more closely related to the progression of ASD.

Guo et al. [16] used a finite element model to determine the vertical load applied when the degenerated disc height was reduced by 33% and the pressure on the disc was increased by 6.1%. The study showed that with the increase in the degree of degeneration of adjacent segments, the pressure on the annulus fibrosus and nucleus pulposus gradually increased, but the activity of the adjacent segments gradually decreased in all directions. Increased pressure on the annulus fibrosus and nucleus pulposus inevitably leads to the acceleration of disc degeneration. Although the pressure on the intervertebral disc increases, the deformation will theoretically increase; however, as the degree of degeneration increases, the disc height decreases, and the properties of the intervertebral disc change, eventually resulting in decreased intervertebral disc deformability. The reduction of disc deformation will eventually lead to a gradual decrease in activity. It can be seen that as the degree of degeneration increases, although the ROM in the adjacent segment gradually decreases, the degeneration of the intervertebral disc may accelerate. It can also be inferred that before surgery, the proximal degeneration of the proximal segment is more severe, and the greater the pressure after fusion fixation, the more likely ASD will develop. This inference is consistent with the conclusions of the clinical study by Anandjiwala et al [26].

The lumbar degeneration model in this study considers only changes in disc height, intervertebral disc parameters, and intervertebral ligament length. However, the actual ASD includes the formation of a callus, stenosis of the vertebral canal, spondylolisthesis, and tearing of annulus fibrosus. These effects are not taken into account in the model, leading to certain limitations of the model. Second, in the process of disc degeneration, the change in disc height is a continuous process. However, the finite element model cannot describe the changes in all degenerative processes; it can refer only to the previous literature and simulate mild and severe degeneration. Third, the muscles, skin, and other soft tissues are not included in the model, although some of the muscles and skin strengths can be simulated to some extent by applying vertical loads. However, this simplification created a discrepancy between the actual situation and our models.

## Conclusions

The degeneration of adjacent segments may be related to the increases in the ROM and IDP of adjacent segments. However, as the degeneration of the intervertebral discs in adjacent segments increases, the ROM of adjacent segments gradually decreases, while the nucleus pulposus and annulus fibrosus pressures gradually increase. Degeneration of the proximal adjacent segment is a risk factor for the degeneration of adjacent segments after lumbar fusion.

## Abbreviations

ASD: Adjacent segment degeneration
IDP: Intervertebral disc pressure
PLIF: Posterior lumbar interbody fusion
ROM: Range of motion
